# Searching for immune correlates in Lassa vaccine development – workshop report

**DOI:** 10.1038/s41541-026-01452-6

**Published:** 2026-05-04

**Authors:** Trevor Brasel, Polina Brangel, Ifedayo Adetifa, Sylvain Baize, David Benkeser, Antonio Bertoletti, Alexander Bukreyev, Sue Charlton, Jakob Cramer, Robert W. Cross, Miles P. Davenport, Devy Emperador, Pierre Formenty, Dean Follmann, Robert F. Garry, Peter B. Gilbert, Sarah C. Gilbert, Jimmy D. Gollihar, Nicholas C. Grassly, Marion Gruber, Swati B. Gupta, Stephan Günther, Adam Hacker, Catherine Hoath, Michael R. Holbrook, Marian Killip, Deborah King, Henshaw Mandi, Vincent J. Munster, Christinah Mukandavire, Lisa Oestereich, Sylvanus Okogbenin, Paul Oloo, Slobodan Paessler, Stanley Plotkin, Laura Richert, David Safronetz, Erica Ollmann Saphire, Alessandro Sette, Amy Shurtleff, Julia Granerod, David Wohl, Marija Zaric, Katrin Ramsauer, Christine Dahlke

**Affiliations:** 1 Coalition for Epidemic Preparedness Innovations (CEPI), Oslo, Norway; 2 FIND, Geneva, Switzerland; 3 Institut Pasteur, Paris, France; 4 Department of Biostatistics and Bioinformatics, Rollins School of Public Health, Emory University, Atlanta, GA USA; 5 Emerging Infectious Diseases Programme, Duke-NUS Medical School, Singapore, Singapore; 6 Departments of Pathology and Microbiology & Immunology, University of Texas Medical Branch (UTMB), Galveston, TX USA; 7 UK Health Security Agency (UKHSA), Chapel Hill, UK; 8 Kirby Institute, University of New South Wales, Sydney, Australia; 9 World Health Organization, Geneva, Switzerland; 10 National Institute of Allergy and Infectious Diseases (NIAID), Bethesda, MD USA; 11 Tulane School of Medicine, New Orleans, LA USA; 12 Fred Hutchinson Cancer Center, Seattle, WA USA; 13 Nuffield Department of Medicine, Pandemic Sciences Institute, Oxford University, Oxford, UK; 14 Department of Pathology & Genomic Medicine, Houston Methodist Research Institute, Houston, TX USA; 15 Department of Infectious Disease Epidemiology, Imperial College London, London, UK; 16 International AIDS Vaccine Initiative (IAVI), New York, NY USA; 17 Bernhard Nocht Institute for Tropical Medicine, Hamburg, Germany; 18 Integrated Research Facility at Fort Detrick, National Institute of Allergy and Infectious Diseases, National Institutes of Health, Frederick, MD USA; 19 Wellcome Trust, London, UK; 20 Division of Intramural Research, Rocky Mountain Laboratories, Laboratory of Virology, National Institute of Allergy and Infectious Diseases, National Institutes of Health, Hamilton, MT USA; 21 University of Hamburg, Hamburg, Germany; 22 Irrua Specialist Teaching Hospital, Irrua, Nigeria; 23 University of Pennsylvania, Philadelphia, PA USA; 24 INSERM, Bordeaux Population Health Research Center, UMR1219, Inria Sistm and CHU de, University of Bordeaux, Bordeaux, France; 25 Inserm, Bordeaux, France; 26 Public Health Agency of Canada, Winnipeg, MB Canada; 27 Center for Autoimmunity and Inflammation, La Jolla Institute for Immunology, La Jolla, CA USA; 28 Department of Medicine, University of California San Diego, La Jolla, CA USA; 29 Dr JGW Consulting AS, Sandefjord, Norway; 30 UNC School of Medicine, Chapel Hill, NC USA

**Keywords:** Computational biology and bioinformatics, Diseases, Immunology, Microbiology

## Abstract

The first workshop dedicated to Lassa virus–specific correlates of protection (CoP) was held in 2024 and was convened by the Coalition for Epidemic Preparedness Innovations (CEPI). Experts from multiple disciplines reviewed existing knowledge and identified gaps in understanding Lassa virus- and vaccine-induced immune responses. Discussions covered key areas including epidemiology, immunogenicity, preclinical and clinical research, data science, and regulatory considerations, with the goal of pinpointing opportunities to discover CoP.

## Introduction

### Importance of Lassa virus-specific immune correlate of protection (CoP)

Lassa fever (LF) is a major public health issue in endemic areas of West Africa, causing 100,000–300,000 infection and approximately 5000 deaths yearly^1,2^. These estimates remain imprecise, as disease surveillance is not conducted consistently or uniformly across settings^3^. LF is an acute hemorrhagic fever caused by Lassa virus (LASV), first isolated in Nigeria in 1969^4,5^. The main reservoir is the peri-domestic rodent, *Mastomys natalensis*^6^. LF is often undiagnosed and symptoms are highly unspecific, mimicking those of other common endemic illnesses including malaria. Among symptomatic infections, 15–20% are severe, with older age and pregnancy associated with increased risk^7,8^. One important sequalae from LASV-infection is hearing loss. There are currently no approved therapeutics or vaccines; all drugs used to treat LF are off-label, thus emphasizing the importance of a vaccine. Vaccine development has been identified as a priority by the World Health Organization (WHO)^9,10^ and CEPI. The urgency of advancing LASV vaccine development is also reflected in the WHO 2024 Pathogens Prioritization Framework, which designates LASV as both a priority pathogen and the prototype pathogen for the *Arenaviridae* family, with a high risk of causing a Public Health Emergency of International Concern (PHEIC)^11^. Several vaccines in the pre-clinical stage or early phase clinical trials are in the pipeline.

A CoP is an immunological biomarker endpoint that can reliably infer vaccine efficacy against a specific outcome by measuring an immune response that reduces the risk of infection or disease. Here, the term “endpoint” is intended to reflect the potential functional role of a CoP in guiding vaccine development and should not be interpreted as implying regulatory qualification. The establishment and regulatory acceptance of a CoP is an iterative process, informed by the totality of available evidence and evaluated within a defined context of use. A CoP may serve as a surrogate endpoint in clinical trials. A correlate of risk (CoR) is an immune marker that is statistically associated with the likelihood of infection or disease in vaccinated or unvaccinated individuals, but it does not necessarily imply causation. A CoR may help identify individuals at higher or lower risk, but it cannot independently infer vaccine efficacy. Both CoPs and CoRs play critical roles in vaccine development.

CoRs can serve as candidate CoPs, highlighting immune markers and assays that should be prioritized for future studies. CoPs can enable comparison of immunogenicity across vaccine candidates, support bridging between populations, provide an essential regulatory tool for emergency use authorization during outbreaks, and facilitate evaluation and potential licensure pathways for routine use in endemic areas where large-scale efficacy trials may not always be feasible due to geographic, temporal, epidemiological, or ethical constraints. A CoP can de-risk phase 3 trials and reduce development time and cost. Identifying a LASV CoP has been recognized as a research priority to advance LF countermeasures by 2030^9^.

CEPI is a global partnership that accelerates vaccine development against epidemic-prone pathogens by financing R&D and convening academic, industry, and regulatory stakeholders to address critical scientific and translational challenges. In its role as a global enabler of vaccine development for epidemic-prone pathogens, CEPI brings together academic, industry, and regulatory experts to align scientific evidence and accelerate translational progress. Seventy-one global experts from 14 countries and diverse scientific, clinical, regulatory and public health disciplines were invited to discuss current knowledge regarding LASV immunology and identify knowledge gaps, challenges, and opportunities to better understand protective immune responses and to advance the discovery of an immunological CoP. Here, we elaborate on discussions during the workshop and ways forward to accelerate the search CoRs and CoPs (see Table 1).

**Table 1:** Gaps and recommendations related to Lassa fever correlates of protection identified during the 2024 workshop

Area	Identified gaps	Recommendations
Cross-functional	*Standardization and harmonization* • Lack of harmonized protocols for sample collection, processing methods and immunological assays across laboratories • Lack of standardized reference panels, viral reagents, and qualified assays *Quality* • Limited implementation of quality management systems and reproducibility frameworks • Inconsistent quality control measures *Infrastructure and capabilities* • Lack of adequate infrastructure (i.e., facilities, equipment, space, etc.) and capabilities, including appropriately trained personnel	*Strengthen the use and access to standardized protocols and reagents* • Create laboratory network(s) and processes to share guidelines, SOPs and reagents • Promote data sharing and meta-analysis • Increase investment for the development of reagents and assays • Where applicable, implement use of WHO International Standards, other gold standards, and lineage-diverse panels *Ensure harmonization across laboratories and improve quality* • Harmonize assays, endpoints, reagents, standards, and protocols across preclinical and clinical programs • Harmonize analytical methods to reduce variability and strengthen comparability • Validate process tools, laboratory equipment, systems and data templates across laboratories • Implement quality management systems and GxP training where needed *Strengthen infrastructure and capabilities* • Invest in adequate infrastructure, including appropriate space and critical equipment • Implement structured training programs, including proficiency testing
LASV-induced immune responses	*Cell-mediated immunity (CMI)* **•** Poor understanding of the role of CMI in protection (breadth, durability, function, poly-functionality) *Humoral immunity* • Limited understanding of the role of non-neutralizing antibodies *Cross-lineage immunity* • Limited understanding of cross-lineage immunity and reinfection protection	*Strengthen human immunology evidence* **•** Conduct longitudinal immunology studies in LF patients (e.g., ENABLE 1.0 /1.5) to assess re-infection, long-term immunity, cross-reactivity (LASV lineages) and prognostic immune markers, but also innate immune responses during acute and recovery phases • Determine mechanisms CMI to LASV and understand their function, role and longevity • Expand research into B-cell and antibody functionality • Characterize non-neutralizing antibody responses and evaluate Fc-mediated functions (e.g., ADCC, NK-cell activation, etc.)
Animal models	*Biological and experimental limitations* • Non-natural infection routes commonly used (intraperitoneal [rodents] and intramuscular [NHP]) • CoP identification hindered by high vaccine efficacy, limited use of uniformly virulent strains, and lack of serum-transfer or dose-down studies	*Advance model development and biological relevance* • Prioritize development of small animal models (e.g., refined guinea pig models), including models that incorporate alternative challenge routes (e.g., mucosal) to better mimic normal route of human infection • Advance preclinical studies on hearing loss • Deepen use of contemporary LASV strains in preclinical studies *Enable CoP and breakthrough studies* • Develop and characterize models that allow for post-vaccination disease breakthrough and challenge models that use more contemporary and/or virulent LASV strains (e.g., Lineage VII) • Implement serum-transfer studies and dose-down studies
Assays	*Commercial products* • Zalgen ReLASV® production discontinued *Assay performance and optimization* • High variability in assay performance due to lineage/epitope differences • ELISAs and neutralization Ab assays need optimization per viral lineage to ensure sensitivity, specificity, and cross-lineage comparability • T-cell assays remain difficult to standardize and challenging to implement consistently across labs *Capacity* • Limited laboratory capacity to perform T-cell assays for Lassa virus due to equipment deficiencies and training requirements	*Commercial products* • Investigate additional commercial assay products (e.g., Panadea LASV ELISA kits) *Assay performance* • Finalize and scale up validated ELISAs (e.g., Panadea) • Simplify and harmonize T-cell assays including collection, thawing, peptide pools and antigens *Expand and build sustainable capacity* • Expand local diagnostic capacity and robust assays in endemic areas • Invest in assays that can be used in lower biocontainment settings, particularly in endemic regions • Develop more user-friendly assays • Expand training of local laboratory staff on best practices for selected assays
Vaccine-induced immune response	*No clear CoP for vaccine candidates identified* • Difficulty interpreting vaccine response due to assay variability • Poor cross-study comparability due to inconsistent platforms, study designs, assays, etc. • Limited data sets	• See cross-functional recommendations
Data science	• Need for optimized trial design • Lack of integration between epidemiology and immunogenicity data	*Apply advanced data-science methods to design efficient, adaptive trials* • Use real-time data to inform trial endpoints and sample size during implementation • Prioritize CoP identification by potentially overpowering trials to ensure enough breakthrough cases for robust analysis **•** Integrate immunological, clinical, and epidemiological data for modeling
Regulatory	• Uncertainty around use of CoP-based approval and indication expansion • Lack of clarity on bridging requirements for new vaccines	*Strengthen early and continuous regulatory engagement* • Engage early with regulators in endemic countries to align expectations and pathways • Work closely with public health officials in endemic countries to understand risk tolerance for expanded use or licensure based on CoPs • Advance collaboration across stakeholders to co-develop coherent regulatory strategies *Define clear requirements for licensure* • Define acceptable immunogenicity and safety datasets to support licensure decisions • Conduct benefit-risk assessment for use of CoP for Lassa fever vaccines *Promote CoP-based regulatory pathways* • Advocate for regulatory frameworks that enable CoP-based decisions • Prioritize generation of robust CoP evidence to underpin these pathways

## Workshop content

### LASV

LASV is an arenavirus with seven known lineages (I-VII)^12^. Its two RNA segments encode four proteins: nucleoprotein (NP), glycoprotein complex (GPC), large protein (L), and zinc-binding (Z) protein^13^. GPC is the key protein that mediates viral attachment and cell entry^14,15^ and is a prime target for vaccines and therapeutics, but is heavily glycosylated, hindering development^16^. In its mature form, GPC consists of GP1, GP2, and a stable signal peptide (SSP) that supports GPC trafficking and triggers membrane fusion^14,17^.

### Immune responses to LASV

LASV infection elicits immune responses, with some individuals controlling the virus while others do not. Factors that influence disease outcome are not well understood nor are protective immune responses against infection and/or re-infection, disease, or mortality. Both humoral and cell-mediated responses are assumed to be critical to generate protection against LASV infection and disease^18–20^. Neutralizing antibody responses are often minimal or undetectable during the acute phase of LF and increase slowly only after convalescence, whereas T cells (both CD4⁺ and CD8⁺) are activated early during infection and remain detectable in patients following recovery^18,20^.

LASV infection elicits humoral immunity targeting NP and GP. IgM NP antibodies are generally detected by day 6 of illness and wane by approximately six months post-disease onset, while IgG NP antibodies can persist for at least 60 months (S Günther, personal communication). In comparison, antibodies against GP appear typically around 3 months post disease onset and gradually decline over time (S. Günther).

Neutralizing antibodies (nAbs) usually appear during the third month post-acute phase when LASV viral clearance and recovery have occurred, reach peak concentrations around month twelve, then slowly decrease^18,20^. The detected delayed nAb response may reflect limited class-switching which may contribute to prolonged IgM persistence^21^. Workshop discussions underscored that natural infection may present GP epitopes in conformations that promote the development of highly potent nAbs. Supporting this, highly protective nAbs isolated from survivors have been shown to recognize properly formed trimer^22^. Studies on GP immunogen design emphasize the need for complete GP cleavage to produce the properly condensed form essential for elicitation of nAbs, shown for protein-based vaccines^23^. GP can be engineered into a form that presents the fully cleaved and condensed pre-fusion conformation that is recognized by nAbs. However, the optimal antigen configuration may vary depending on the vaccine platform.

It is important to distinguish nAbs responses from binding Abs or Fc-mediated functions (non-neutralizing Abs), which may follow different kinetics and could contribute to protection through mechanisms not captured by classical neutralization assays. The relative contribution of antibody subclasses and functions to protection is not well understood and remains an active area of investigation. Consequently, the overall contribution of antibodies, including nAbs, to LASV clearance and protection from severe disease following infection is not yet clearly defined. Harmonized assays will be essential for comparing findings across natural infection, vaccine studies, and animal models.

T-cells likely play a role in pathology and recovery from illness. CD4+ and CD8+ T cells are detectable against NP and GP post-infection. It is assumed that specific T-cell responses contribute significantly to viral clearance and recovery. Severe disease and fatality have been associated with a predominance of non-specific, bystander T-cell activation with homing of these cells into inflamed tissue. Mild disease and recovery are associated with activation of predominantly specific effector T-cells^24^.

During LASV infection, NP constitutes an immunodominant target of CD8+ T-cell responses, whereas GPC predominantly elicits binding and neutralizing antibody responses and is comparatively less prominent in CD8+ T-cell recognition compared to NP-directed responses. LaVergne et al. highlighted that LASV survivors retain long-term CD8+ T-cell memory detectable after in vitro expansion with responses largely focused on NP, while GPC contributes comparatively lower recognition to CD8+ T-cell recognition^25^.

Workshop recommendations emphasize the need to evaluate humoral immune response data with emerging evidence on T‑cell and innate immune responses to develop a more comprehensive understanding of CoP. Critical knowledge gaps remain regarding the nature, breadth, and durability of protective immunity following infection with LASV. It is unclear whether immunity against LF upon re-exposure is complete, partial, or lineage-dependent. The extent to which immune responses generated against one viral lineage confer cross-protection against other genetically diverse lineages remains insufficiently characterized. While no cases of repeated LF have been observed in hospitals (per informal reports), there is no evidence to confirm this. Ongoing studies are essential to assess long-term immunity and re-infection risk^26^. Studies like ENABLE 1.0/1.5 will be critical to evaluate and better understand long-lasting immunity against LASV following infection and the risk of re-infection^27^.

### Animal models

Animal models can advance our knowledge of human symptoms, pathogenesis, and sequelae, as well as for elucidating humoral and cellular immune responses. The ideal LF animal model, one that fully recapitulates disease and pathogenic features of LF in humans, does not exist^28^. Current preferred animal models include mouse, guinea pig, and non-human primates (NHP) (Table 2); further insight into reservoir host responses and transmission has been gained through studies conducted in more non-traditional models, including the natural LASV host *Mastomys natalensis*^28^. Limitations must be recognized, and not all findings can be extrapolated to humans.

**Table 2:** Current preferred LASV animal models

Animal model	Strain	LASV challenge model description
Mouse	STAT1^−/−^	No virus adaptation needed to establish disease. Infection results in morbidity/mortality, body weight loss, systemic infection, and LASV-induced hearing loss. Disease severity depends on virus strain/lineage and challenge route. Key limitations: (1) lack of functional STAT1 pathway (essential for Type I and Type II IFN signaling) may lead to underlying disease mechanisms that do not reflect natural progression of LF in humans; (2) incompatible with many live attenuated vaccines; (3) correlates of protection in immunocompromised mice may not align with those required for a protective human vaccine
	IFNAR^−/−^	No virus adaptation needed to establish disease. Infection results in morbidity/mortality, body weight loss, and systemic infection. Disease severity depends on virus strain/lineage and challenge route. Key limitations: (1) lack of Type 1 IFN signaling leads to a severely impaired innate immune response which carries over into the adaptive immune response as IFNs are crucial for properly activating B cells and T cells; (2) incompatible with many live attenuated vaccines; (3) lower lethality compared to STAT1^−/−^ mice; (4) correlates of protection in immunocompromised mice may not align with those required for a protective human vaccine
Guinea pig	Strain 13	No virus adaptation needed to establish disease. Infection results in morbidity/mortality, body weight loss, fever, liver damage, and respiratory distress. Disease severity depends on virus strain/lineage and challenge route. Limited availability restricts widespread use. Key limitations: (1) lack of commercial availability; (2) often develop myocarditis or endocarditis not typically seen in human LF; (3) lack of guinea pig-specific immunological reagents
	Hartley	Most studies have shown that virus adaptation is needed to establish disease, but recent studies have shown that some contemporary LASV isolates can induce disease without adaptation. Infection results in morbidity/mortality, body weight loss, fever, lethargy, and respiratory distress. Disease severity depends on virus strain/lineage and challenge route. Readily available. Key limitations: (1) lack of guinea pig-specific immunological reagents; (2) often develop pulmonary edema and myocarditis not typically seen in human LF; (3) due to being outbred, individual Hartley guinea pigs may show highly variable clinical and immunological responses; while this mimics human diversity, larger group sizes may be required to achieve statistical significance
Nonhuman primate	Cynomolgus macaque (*Macaca fascicularis*)	No virus adaptation needed to establish disease. Infection results in morbidity/mortality, body weight loss, fever, rash, facial edema, lethargy including hunched posture, dehydration, respiratory distress, neurological signs, hearing loss/deafness. Disease severity depends on virus strain/lineage and challenge route. Key limitations: (1) supply shortage, expense, and ethical considerations; (2) disease course may vary depending on origin; (3) higher susceptibility to LASV infection than humans
	Rhesus macaque (*Macaca mulatta*)	No virus adaptation needed to establish disease. Infection similar to that described for cynomolgus macaques, although severity is often defined as less severe based on published results demonstrating decreased lethality. Key limitations: (1) supply shortage, expense, and ethical considerations; (2) may demonstrate resistance to lethal LF; (3) may demonstrate hyper immune responses including broader and stronger T-cell responses; (4) MHC polymorphism

Wild-type mice are predominantly resistant to LASV^29^, while some immunocompromised models mimic certain aspects of human LF. Studies in STAT1^−/−^ mice have linked LASV-induced hearing loss to spiral ganglion cell loss, with CD8+ T-cells contributing to disease^30^. Other models, including mice deficient for type I interferon receptor and humanized mice genetically engineered to express human/mouse-chimeric HLA-A2.1, have provided insight into immune responses and for early screening of treatments and vaccines^29^. However, none fully replicate human LF pathophysiology. Further, immunocompromised models are unable to mount a fully intact immune response to vaccines, necessitated caution when interpreting results. Together, these aspects highlight the need for further model refinement.

Guinea pig (GP) models of LASV infection share several disease characteristics of LF in humans. Historically, two GP models, Strain 13 and Hartley, have been used in preclinical studies^28,31–33^. Strain 13 GPs are inbred and support disease establishment without the need for virus adaptation; however, their limited availability restricts widespread use. Hartley GPs are readily accessible from commercial suppliers but typically require virus adaptation. Nevertheless, recent studies have shown that some contemporary LASV isolates can induce disease in Hartley GPs without prior adaptation^34^. In general, GP models are important for testing vaccine platforms or therapeutic interventions^29^.

NHP models of LF recapitulate aspects of human disease and immune responses, making them invaluable for studying pathogenesis, testing vaccines and treatments, and understanding transmission dynamics^28,29,35^. Cynomolgus macaques (*Macaca fascicularis*) may be preferable for testing medical countermeasures that specifically target virus infection and/or replication due to near-uniform lethality^36,37^. During the workshop, it was discussed that depletion of CD4+ or CD8+ T-cells in cynomolgus macaques does not significantly alter the disease endpoint; this depletion model may offer the ability to assess mechanisms of protection afforded by specific vaccines, information which could potentially be used to translate findings to humans. A single installation of monoclonal antibody cocktail, either 14 or 7 days prior to challenge, has been shown to protect NHPs from LF^38^.

### Immunological assays

Immunoassays for monitoring humoral and cell-mediated immunity (CMI) responses to LASV encompass a diverse range of techniques^39^. At the time of the workshop (February 2024), the landscape of commercially available assays for humoral immune responses against LASV included approximately 25 products (Table S1), most of which were produced by one manufacturer. As of December 2024, this manufacturer (Zalgen Labs) has ceased production of LASV-specific assays and reagents. The resulting lack of commercially available products has impacted the field significantly and, to this day, remains a critical unmet need^40^.

Most immunological humoral assays focus on IgM and IgG responses targeting NP and GP. Currently, no gold standard exists for these assays and none have regulatory approval (i.e., they are classified as Research Use Only)^41^. ELISAs to detect binding Abs show strong sensitivity but mixed results concerning specificity. Notably, implementing the first WHO International Standard for anti-LASV antibodies^42^ is crucial for enabling comparability between laboratories and across different assays, thereby enhancing the global reliability and consistency of immune response data. During the workshop, the use of different antigens was also debated, with a preference for using pre-fusion and linked Glycoprotein. Preliminary data suggested that utilizing pre-fusion GP in ELISAs could be a promising strategy, as first data underlined correlation with nAb.

The live LASV plaque reduction neutralization test (PRNT) assay represents the gold standard for evaluating nAbs but poses challenges in execution and validation. A practical alternative is the pseudovirus neutralization assays as live LASV is not used. They are more accessible but must be correlated with the PRNT assay and need robust validation against established gold standards.

Analyzing T-cell responses is challenging and difficult to validate. Several assays can be used, including Interferon Gamma Release Assay (IGRA), Enzyme-linked Immunosorbent Spot (ELISpot), Intracellular Cytokine Staining (ICS) and Activation Induced Marker (AIM) flow cytometry. Each assay has benefits, limitations, and challenges. T-cell analysis is often seen as challenging in resource-limited settings, but new approaches, such as IGRA or rapid cytokine assays, which was used in SARS-CoV-2 studies in rural Kenya, show that CMI can be assessed without PBMC isolation, sophisticated equipment, or large blood volumes^43,44^. These assays work with fresh whole blood, simplifying logistics^45^ and allow for cytokine quantification, providing valuable functional insights into immune responses^44^. Although the type and number of cytokine-secreting cells cannot be measured, it remains useful for detecting cytokine responses in infected or vaccinated individuals^43^.

Lastly, the importance of establishing laboratory networks (like the CEPI Centralised Laboratory Network [https://cepi-tr.tghn.org/research-and-development/centralized-laboratory-network/]) with delivering standardized testing but also ensuring validated digital tools was stressed to ensure consistent assay performance and consistent monitoring of results across sites.

### Vaccine-induced immune responses

There are currently no vaccines licensed to prevent LF. However, significant progress was made in LF vaccine development over the last decade, with the first vaccine candidates entering clinical trials in 2019. Several vaccine platforms, including viral vectors (e.g., Mopeia, rVSV, and ChAdOx), DNA and mRNA vaccines, and protein-based vaccines, showed immunogenicity and efficacy in GP and NHP models^12^. Although early experimental LASV vaccine candidates expressing NP and GPC included vaccinia virus–vectored constructs, current clinical-stage candidates are largely based on platforms such as mRNA and adenoviral vectors. These candidate vaccines have all shown 100% protection in GPs in the absence of measurable nAB responses^46–50^. During the workshop, experts discussed preclinical and clinical data from advanced LASV vaccine candidates. Additional insights on current Lassa virus vaccine candidates are discussed in published reviews^51,52^.

An N1-methylpseudouridine-modified LASV mRNA vaccine encapsulated in lipid nanoparticles that encodes wild-type LASV GPC or the prefusion stabilized GPC has been shown to protect against LASV disease in the guinea pig model despite a lack of detectable nAbs^46^. Further, two live-attenuated viral vector vaccine candidates were discussed: one is based on the recombinant Mopeia virus (MOPEVAC) and the other on a measles-vector, MV-LASV. Both underscore the pivotal role of T-cells in providing protection^53,54^. During preclinical and Phase 1 studies, MV-LASV was shown to induce memory T-cell responses^30^. Cynomolgus macaques vaccinated with MV-LASV or MOPEVAC (Lineage IV) demonstrated robust cross-reactive T-cell responses^55^.

Data from pre-clinical trials of a replication-competent rVSV-LASV vaccine candidate (Lineage IV GPC) showed 100% efficacy as shown by protection against death and symptomatic LF disease in NHPs even when vaccinated one year prior to a lethal homologous LASV challenge^37,56^. A Phase 1 trial with this vaccine has shown a safe and immunogenic profile^57^. The vaccine induced anti-LASV-GP binding (IgG, IgM) and neutralizing antibodies in healthy adult volunteers. Antibodies maintained for at least one year detectable. The vaccine expands polyfunctional T-cell responses.

The ChAdOx1-Lassa-GPC vaccine underlined a critical CMI response with detected cross-reactive T-cell responses across lineages in vaccinated mice^47,58^. In GP, the vaccine provided protection with one or two doses against homologous (lineage IV) and heterologous (lineages I–IV) LASV challenge^47^. In cynomolgus macaques, at least 70% of vaccinated animals were protected from morbidity and 100% from mortality^59^. In these animals, LASV IgG levels rose and remained stable until challenge while nAbs were minimal and lacked correlation with protection suggesting a key role for T-cells.

### Data science

Trial design, data science, and statistical analysis are key for identifying CoP against infection, disease, or mortality. The two tiers of data analysis for assessing immune correlates in a vaccine efficacy trial include CoR and CoP^60,61^. CoR estimates the association between immune marker and clinical outcome in vaccinees, while CoP estimates parameters linking vaccine efficacy to the immune marker. Compelling CoP results come from population-level analyses, which require harmonization of vaccine efficacy trials in terms of study design, immunoassays, and statistical analysis. Vaccine efficacy trials need to collect prognostic factors that may confound the association of immune marker levels with outcome or that may moderate CoRs or CoPs.

For many viruses, nAb titers (and binding antibody concentrations) are strong inverse CoRs and accepted as CoPs for use in specific applications. While these assays may not fully explain the protective effects of a candidate vaccine, they could therefore demonstrate sufficient predictive capabilities to serve as a practical CoP (that may be non-mechanistic) for applications including immuno-bridging to provisionally or fully approved vaccines or extend vaccine indications to new populations. Prioritizing these classical assays for LASV could be advantageous for emergency and non-emergency use given their promise for success and the feasibility of rapid, standardized CoP development. However, these assays may not be sufficient or sensitive enough for all candidate vaccines, as some vaccines can show protective efficacy in animal models without inducing detectable nAbs (e.g., ChAdOx). In addition, detectable immune responses do not always confer protection and/or translate to humans as shown by some vaccine candidates. Multiple factors may contribute to this including inconsistent immunogenicity data, variable endpoints across studies, and preclinical models that may not fully translate to humans. A case-cohort sampling design would allow for efficient CoP assessment across multiple study endpoints by re-using the same immunogenicity sub-cohort^62–64^.

CoPs may differ by study endpoint requiring the need for separate or integrated assessments by LASV endpoint^65,66^. Protection against infection and disease may rely on different immune markers. In polio, serum antibodies prevent paralysis, while mucosal antibodies block infection at entry points. Neutralizing antibody titers are imperfect indicators as they may miss low-level protective responses. Other immune factors (e.g., T-cells) also contribute, especially against severe disease. For LASV, prioritizing endpoints with clear clinical disease phenotypes helps avoid misclassification, which can significantly bias CoR/CoP analyses.

To achieve statistical precision in the evaluation of CoPs, it is essential and critical to prioritize qualified or validated assays and immune marker readouts that demonstrate substantial inter-individual variability among vaccinees while maintaining low technical measurement error. Assays measuring non-mechanistic CoPs may, in some settings, offer greater reproducibility and scalability when compared to assays that seek to quantify the type of functional immune responses that are characteristic of mechanistic CoPs^67–69^. Identification of mechanism of action may be supportive, though a complete understanding of the mechanism of action is not a regulatory requirement if association to protection and assay performance are robust.

Immunophenotype sieve analysis is especially powerful for CoP assessment because it is based directly on the CoP assay readout and the virological genotyping assay; however, it is resource-intensive and a sequence-predicted version of immunophenotype sieve analysis may be more feasible. It has to be acknowledged that the decision to optimize all known parameters is not always the way to choose. In some cases, there is a need to identify new factors that can be used as correlates. One example would be using T-cells as correlate, which has been often suggested as possible correlate but is challenging to be included in large clinical trials. In general, serological correlates have practical advantages, as antibody assays are generally easier to standardize, require less complex sample handling, and are more readily transferable across laboratories. In addition, humoral correlates can provide mechanistic evidence of protection in experimental settings, for example, through passive transfer studies. Despite extensive investigation of T-cell correlates for pathogens such as influenza and COVID-19, regulatory approval pathways have historically relied on antibody-based correlates where available. However, for LASV, available evidence suggests that cellular immune responses play a critical role in protection, and serological markers alone may not fully capture protective immunity.

Rigorous identification of immune CoP against LF disease in humans is likely to require very large studies unless enrollment is highly targeted or reactive. Peak and exposure proximal correlates may be useful and complementary. Test-negative immune correlate analysis^70^, which involves sample collection at the time of case identification from LF test-positive cases vs. test-negative controls, can be more efficient than prospective correlates studies (hundreds of samples versus thousands). Test negative type designs may also allow efficient evaluation of T-cell and other immune correlates because the number of samples to be tested is substantially lower than in a prospective cohort design. However, delays in sample collection and evolving immune response in cases may undermine the relevance of test-negative design assessments of CoP against LASV infection or disease. And notably, case detection of LF is challenging as LASV infection can be asymptomatic.

### Regulatory perspectives

Vaccine-associated biomarkers typically include measures of immune responses that serve either as a scientifically well-established biomarker for protection from disease or as a surrogate endpoint reasonably likely to predict benefit. Identification of biomarkers/CoPs relies on clinical efficacy endpoints that link immune markers to demonstrated protection against disease. Where direct efficacy data are not available, such as under immunobridging or other alternative licensure pathways, immune markers are considered only when supported by prior evidence demonstrating a reasonable likelihood of predicting clinical benefit, informed by clinical data and/or well-characterized, biologically relevant animal models within a defined context of use. From a regulatory perspective, biomarkers can be used for many different objectives. The *context of use* is the key factor that defines how a biomarker can be applied in a specific regulatory setting. Some caveats exist in using biomarkers to infer vaccine effectiveness and support approval. First, the applicability of the biomarker may depend on vaccine characteristics (e.g., antigen structure, mode of delivery) and vaccine platform. In addition, biomarkers require the availability of validated assays for reliable measurement. Accordingly, immunogenicity data should be interpreted with caution, and candidate correlates must undergo rigorous validation against clinical efficacy endpoints or well-supported surrogate endpoints before being used to inform vaccine development or regulatory decision-making.

Once a full-scale efficacy study has been conducted for a LASV-specific vaccine and licensure has been granted based on safety, efficacy, and immunogenicity data, if a biomarker or CoP is identified and validated, it can then be used for establishing duration of protection or expanding the indication to additional populations. In addition, other developers could conduct safety and immunogenicity studies and leverage the CoP to seek licensure based on immunogenicity studies bridging to the licensed product. CoP may not be applicable beyond the vaccine and the population in which they were identified, and scientific understanding and justification are needed to support accelerated registration pathways. Ultimately, a benefit-risk assessment for the use of CoP for LF vaccines is needed. It is critical to work closely with public health officials in endemic countries to understand risk tolerance for expansion of use or registration of new vaccines leveraging CoP.

In brief, early and sustained regulatory engagement, particularly in endemic countries, will be critical to establish how CoP-based evidence may support indication expansion, major manufacturing or process changes, technology transfer, and determination of duration of protection without the need for additional clinical efficacy trials.

Regulatory clarity is also needed on whether, once an initial product is licensed, additional vaccines could be approved through immunobridging to an established CoP rather than clinical endpoint efficacy studies, contingent on assay validation across platforms and formulations. Finally, regulators should specify what additional data, beyond safety and immunogenicity, would be required for other developers to bridge to a licensed vaccine.

## Conclusions

### Recommendations to advance the field

During the workshop, recommendations were emphasized to advance the field of Lassa-specific CoP highlighted in Table 1. Key considerations for implementation of future vaccine trials included early community engagement, Ministry of Health and/or national authorities’ commitment, robust and harmonized trial strategy to evaluate CoP, training and support of local human resources, sustainability of sites, including provision of maintenance, and definition of testing strategy.

A range of options can be considered for laboratory setup, including investment in assay harmonization, strengthening existing infrastructure and personnel, and supporting with mobile laboratories when necessary to expand current capacities. In terms of clinical study design, the identification of measurable immune markers and the use of scalable assays are essential. Pre-existing immunity may confound the assessment of vaccine-induced CoP. Therefore, baseline samples should be collected from all participants and immune responses measured as needed. While it is critical to store baseline blood samples from all participants, it may not be necessary to conduct immunoassays for all participants. A CoP may also be identified following vaccine introduction through cohort studies, provided these are prospectively designed to include sample collection prior to disease onset.

Enhancing data reliability and building confidence in immune markers is a key challenge. To ensure comparability between different sites, it is critical to collect consistent and high-quality data. Strengthening the infrastructure for robust pre-clinical and clinical studies is also necessary, along with sharing protocols, reagents, and data within the community.

Although the workshop identified key evidence gaps and opportunities, it did not address their relative prioritization, which is an essential step for strengthening the evidence base and accelerating progress towards defining a CoP. A systematic prioritization of these gaps will be critical to guide future research investments, support informed funding decisions, and shape the design of subsequent studies.

To advance the Lassa CoP field, several initiatives are already underway, including CEPI-led efforts such as the CLN and PMN, the development of standardized reagents and assays, the generation of new immunological and clinical datasets, and the development of evidence maps, guidelines, gap analyses, and targeted recommendations through a CoP Playbook. Alignment and consistency across these activities will be essential to ensure comparability of datasets and to build confidence in identified immune markers.

## Supplementary information

Supplementary Information

## Data Availability

No datasets were generated or analyzed during the current study.
