# Supplementary material for: Searching for immune correlates in Lassa vaccine development – workshop report
Source: NPJ Vaccines. 2026 May 4;11:95. doi: 10.1038/s41541-026-01452-6 (PMC13136350; doi:10.1038/s41541-026-01452-6)
Supplement: Supplementary file 1 — Supplementary Information [file 41541_2026_1452_MOESM1_ESM.pdf]

Website	Assay name	Assay target	Stage of development	Regulatory body	Technology principle	Instrument requirement	Laboratory/ Point of care	Self-testing or professional use	Validated sample types	Manufacturer	Country of manufacturer HQ	Region of manufacturer HQ
https://sunlongbiotech.com	Human Lassa Virus IgM ELISA Kit	Antibody (serological)	Research Use Only (RUO)	None	ELISA/EIA	Standard lab instrumentation is required	Lab-based	Intended for professional use only	Serum;Plasma;Urine; Tissue/Biopsy;Other	Sunlong Biotech	China	Western Pacific Region
https://zalgen.com	ReLASV Pan-Lassa Combo NP/ Prefusion GP IgM ELISA Kit – RUO	Antibody (serological)	Research Use Only (RUO)	None	ELISA/EIA	Standard lab instrumentation is required	Lab-based	Intended for professional use only	Serum;Plasma	Zalgen Labs	United States	Region of the Americas
https://zalgen.com	ReLASV Pan-Lassa Combo NP/ Prefusion GP IgG ELISA Kit – RUO	Antibody (serological)	Research Use Only (RUO)	None	ELISA/EIA	Standard lab instrumentation is required	Lab-based	Intended for professional use only	Serum;Plasma	Zalgen Labs	United States	Region of the Americas
https://zalgen.com	ReLASV Pan Lassa NP IgM ELISA Kit RUO	Antibody (serological)	Research Use Only (RUO)	None	ELISA/EIA	Standard lab instrumentation is required	Lab-based	Intended for professional use only	Serum;Plasma	Zalgen Labs	United States	Region of the Americas
https://zalgen.com	ReLASV Pan Lassa Prefusion GP IgM ELISA Kit RUO	Antibody (serological)	Research Use Only (RUO)	None	ELISA/EIA	Standard lab instrumentation is required	Lab-based	Intended for professional use only	Serum;Plasma	Zalgen Labs	United States	Region of the Americas
https://sunlongbiotech.com	Human Lassa Virus IgG ELISA Kit	Antibody (serological)	Research Use Only (RUO)	None	ELISA/EIA	Standard lab instrumentation is required	Lab-based	Intended for professional use only	Serum;Plasma;Urine; Tissue/Biopsy;Other	Sunlong Biotech	China	Western Pacific Region
https://bnitm.de	BLACKBOX LASV IgG ELISA Kit RUO	Antibody (serological)	Research Use Only (RUO)	None	ELISA/EIA	Standard lab instrumentation is required	Lab-based	Intended for professional use only	Serum	Bernhard Nocht Institute for Tropical Medicine	Germany	European Region
https://bnitm.de	BLACKBOX LASV IgM ELISA Kit RUO	Antibody (serological)	Research Use Only (RUO)	None	ELISA/EIA	Standard lab instrumentation is required	Lab-based	Intended for professional use only	Serum	Bernhard Nocht Institute for Tropical Medicine	Germany	European Region
https://lshtm.ac.uk	Lassa IgG Double Antigen Binding Assay (DABA) ELISA	Antigen	Research Use Only (RUO)	None	ELISA/EIA	Standard lab instrumentation is required	Lab-based	Intended for professional use only	Saliva;Serum;Plasma; Other	London School of Hygiene & Tropical Medicine Hospital for Tropical Diseases	United Kingdom	European Region
https://zalgen.com	ReLASV Pan-Lassa Antigen Rapid Test RUO	Antigen	Research Use Only (RUO)	None	Lateral Flow Assay	No instrumentation is required	True Point of Care	Intended for professional use only	Serum;Plasma;Finger prick blood;Whole Blood	Zalgen Labs	United States	Region of the Americas
https://zalgen.com	ReLASV Pan Lassa NP IgG/IgM ELISA Kit RUO	Antibody (serological)	Research Use Only (RUO)	None	ELISA/EIA	Standard lab instrumentation is required	Lab-based	Intended for professional use only	Serum;Plasma	Zalgen Labs	United States	Region of the Americas
https://zalgen.com	ReLASV Pan Lassa Prefusion GP IgG/IgM ELISA Kit RUO	Antibody (serological)	Research Use Only (RUO)	None	ELISA/EIA	Standard lab instrumentation is required	Lab-based	Intended for professional use only	Serum;Plasma	Zalgen Labs	United States	Region of the Americas
https://zalgen.com	ReLASV Pan Lassa Linked GP IgG/IgM ELISA Kit RUO	Antibody (serological)	Research Use Only (RUO)	None	ELISA/EIA	Standard lab instrumentation is required	Lab-based	Intended for professional use only	Serum;Plasma	Zalgen Labs	United States	Region of the Americas
https://zalgen.com	ReLASV Pan Lassa Prefusion GP IgG ELISA Kit RUO	Antibody (serological)	Research Use Only (RUO)	None	ELISA/EIA	Standard lab instrumentation is required	Lab-based	Intended for professional use only	Serum;Plasma	Zalgen Labs	United States	Region of the Americas
https://zalgen.com	ReLASV Pan Lassa Combo NP/ Prefusion GP IgG/IgM ELISA Kit RUO	Antibody (serological)	Research Use Only (RUO)	None	ELISA/EIA	Standard lab instrumentation is required	Lab-based	Intended for professional use only	Serum;Plasma	Zalgen Labs	United States	Region of the Americas
https://zalgen.com	ReLASV Combo NP/PF GP IgG ELISA Kit RUO	Antibody (serological)	Research Use Only (RUO)	None	ELISA/EIA	Standard lab instrumentation is required	Lab-based	Intended for professional use only	Serum;Plasma	Zalgen Labs	United States	Region of the Americas
https://zalgen.com	ReLASV NP Lineage II IgG ELISA Kit RUO	Antibody (serological)	Research Use Only (RUO)	None	ELISA/EIA	Standard lab instrumentation is required	Lab-based	Intended for professional use only	Serum;Plasma	Zalgen Labs	United States	Region of the Americas
https://zalgen.com	ReLASV Linked GP Lineage II IgG ELISA Kit RUO	Antibody (serological)	Research Use Only (RUO)	None	ELISA/EIA	Standard lab instrumentation is required	Lab-based	Intended for professional use only	Serum;Plasma	Zalgen Labs	United States	Region of the Americas
https://zalgen.com	ReLASV Linked GP Lineage IV IgG ELISA Kit RUO	Antibody (serological)	Research Use Only (RUO)	None	ELISA/EIA	Standard lab instrumentation is required	Lab-based	Intended for professional use only	Serum;Plasma	Zalgen Labs	United States	Region of the Americas
https://zalgen.com	ReLASV Pan Lassa Linked GP IgG ELISA Kit RUO	Antibody (serological)	Research Use Only (RUO)	None	ELISA/EIA	Standard lab instrumentation is required	Lab-based	Intended for professional use only	Serum;Plasma	Zalgen Labs	United States	Region of the Americas
https://zalgen.com	ReLASV NP Lineage IV IgG ELISA Kit RUO	Antibody (serological)	Research Use Only (RUO)	None	ELISA/EIA	Standard lab instrumentation is required	Lab-based	Intended for professional use only	Serum;Plasma	Zalgen Labs	United States	Region of the Americas
https://zalgen.com	ReLASV Pan Lassa Antigen ELISA Kit RUO	Antigen	Research Use Only (RUO)	None	ELISA/EIA	Standard lab instrumentation is required	Lab-based	Intended for professional use only	Serum;Plasma	Zalgen Labs	United States	Region of the Americas
https://zalgen.com	ReLASV Pan Lassa NP IgG ELISA Kit RUO	Antibody (serological)	Research Use Only (RUO)	None	ELISA/EIA	Standard lab instrumentation is required	Lab-based	Intended for professional use only	Serum;Plasma	Zalgen Labs	United States	Region of the Americas
https://zalgen.com	ReLASV Prefusion GP Lineage II IgG ELISA Kit RUO	Antibody (serological)	Research Use Only (RUO)	None	ELISA/EIA	Standard lab instrumentation is required	Lab-based	Intended for professional use only	Serum;Plasma	Zalgen Labs	United States	Region of the Americas
https://zalgen.com	ReLASV Prefusion GP Lineage IV IgG ELISA Kit RUO	Antibody (serological)	Research Use Only (RUO)	None	ELISA/EIA	Standard lab instrumentation is required	Lab-based	Intended for professional use only	Serum;Plasma	Zalgen Labs	United States	Region of the Americas
